# Cost-effectiveness analysis of cetuximab/irinotecan vs active/best supportive care for the treatment of metastatic colorectal cancer patients who have failed previous chemotherapy treatment

**DOI:** 10.1038/sj.bjc.6603561

**Published:** 2007-01-23

**Authors:** N Starling, D Tilden, J White, D Cunningham

**Affiliations:** 1 Royal Marsden Hospital, Sutton, Surrey, UK; 2 M-TAG Limited, Lee House, London, UK; 3 Merck Pharmaceuticals, UK; 4 Royal Marsden Hosptal, Sutton, Surrey, UK

**Keywords:** cetuximab, colorectal, cost-effectiveness

## Abstract

The treatment of colorectal cancer is rapidly becoming a significant financial burden to health-care systems within economically developed nations. A current challenge for oncologists and health-care payers is to integrate new, often high-cost, biologic therapies into clinical practice. Inherent to this process is the consideration of cost-effectiveness. The aim of this study was to compare the cost-effectiveness of cetuximab plus irinotecan with an appropriate comparator from a National Health Service (NHS) perspective. This economic evaluation is a trial-based study of cetuximab vs active/best supportive care. Effectiveness estimates for the treatment groups were modelled from key clinical trials. Cunningham *et al* (2004) compared cetuximab/irinotecan with cetuximab monotherapy; Cunningham *et al* (1998) compared irinotecan monotherapy in a second-line setting with supportive care. Modelling was necessary owing to an absence of head-to-head clinical trial data of cetuximab/irinotecan vs current standard care. Costs were calculated for the study drugs received, associated administration, palliative chemotherapy for patients in the standard care arm and other nonchemotherapy resources. The discounted life-expectancy of patients treated with cetuximab/irinotecan was 0.91 life-years, and 0.47 discounted life-years for patients receiving active/best supportive care. Patients treated with cetuximab/irinotecan accumulated mean additional costs of £18 901 per patient relative to the comparator arm, with £11 802 attributable to cetuximab. The incremental cost per life-year gained with cetuximab/irinotecan therapy compared with active/best supportive care was £42 975. The incremental cost per quality adjusted life-year gained was £57 608. The incremental cost per life-year gained for cetuximab/irinotecan is relatively high compared with other health-care interventions. However, this result should be considered in the context of a number of factors specific to the treated patient population.

Colorectal cancer (CRC) is the third most commonly diagnosed cancer globally (Parkin, *et al*, 2001), with almost 30 000 new cases diagnosed in England and Wales per year, representing more than 12% of new cancers diagnosed. About 30% of patients present with advanced colorectal cancer, defined as either metastatic or locally invasive disease (NICE, 2005). Advances in the treatment of CRC in both the adjuvant and metastatic settings have presented significant financial implications for health-care systems in economically developed countries.

Treatments for metastatic CRC are mainly palliative and aim to increase the duration and the quality of the patient's remaining life. For many years, 5-fluorouracil (5-FU) was the only active agent in colorectal cancer associated with survival in the region of 12 months (Metaanalysis, 1994, 1998). Over the last decade significant progress has been made, as several new chemotherapeutic agents have been incorporated into routine clinical practice. Irinotecan, a semisynthetic inhibitor of topoisomerase, and oxaliplatin, a third-generation platinum compound, were developed as salvage therapies for patients failing 5-FU (Cunningham *et al*, 1998; Rothenberg *et al*, 2003), and are now established treatment options for use as first-line, second-line and sequential treatment in CRC (de Gramont *et al*, 2000; Douillard *et al*, 2000; Saltz *et al*, 2000; Goldberg *et al*, 2004; Grothey *et al*, 2004; Tournigand *et al*, 2004). The notion of sequential treatment as a determinant of survival has become important, with overall survival of over 21 months now achievable (Tournigand *et al*, 2004). The National Institute of Health and Clinical Excellence (NICE) recently approved the sequential use of oxaliplatin and irinotecan-based chemotherapy in the first- and second-line treatment of CRC.

Developments in the cytotoxic management of CRC have progressed in tandem with the clinical development of biologic agents in CRC. Cetuximab is a monoclonal antibody that targets the epidermal growth factor receptor (EGFR), which governs several cellular processes pertinent to tumour development and progression. In preclinical studies modulating EGFR-mediated signalling through antibody inhibition resulted in antitumour activity, the reversal of resistance to irinotecan and synergy with irinotecan (Prewett *et al*, 2002). In clinical evaluation, two phase II studies indicated that cetuximab was active in combination with irinotecan and as monotherapy in irinotecan-resistant patients (Saltz *et al*, 2001; Saltz *et al*, 2004). The Bowel Oncology with cetuximab aNtiboDy study (BOND; Cunningham *et al*, 2004) compared cetuximab in combination with irinotecan against cetuximab monotherapy in heavily pretreated EGFR-expressing patients, recording response rates of 23 and 11% respectively, with a median time to progression favouring the combination arm (4.1 vs 1.5 months, *P*=0.0001) (Cunningham *et al*, 2004). Median survival was not statistically different between the two arms (8.6 vs 6.9 m), which may have been a consequence of crossover. In the BOND study, patients had received a median of three prior treatment regimens; 100% of patients had received irinotecan and 62% of patients had also received prior oxaliplatin. Based on these results, cetuximab was licensed in Europe for the treatment of irinotecan-refractory colorectal cancer in combination with irinotecan in 2005. Given the current NICE guidance for the use of oxaliplatin and irinotecan, the potential application of cetuximab in the UK is likely to be in the third-line setting for which there are currently no standard therapeutic interventions.

The current challenge for oncologists and health-care payers alike lies in the optimal integration of high-cost biologic therapies such as cetuximab into clinical practice. Inherent to this process is the consideration of cost-effectiveness. The aim of this study was to compare the cost-effectiveness of cetuximab treatment with the appropriate comparator for the National Health Service (NHS) (i.e. active/best supportive care). The definition of active/best supportive care (ASC/BSC) is adapted from the description of supportive care in a key trial of active chemotherapy vs BSC in the second-line setting (Cunningham *et al*, 1998). Active supportive care is defined as the best care available, as judged by a physician, and may include chemotherapy. Supportive interventions may include: antibiotics, analgesics, transfusions, corticosteroids, or any other symptomatic therapy and/or assistance of a psychotherapist, and localised radiation therapy (dose within palliative range) to alleviate symptoms. Best supportive care excludes the possibility of active chemotherapy.

## MATERIALS AND METHODS

The economic evaluation presented is a trial-based study of cetuximab/irinotecan vs ASC/BSC. This is an indirect comparison owing to the absence of a direct head-to-head trial of cetuximab versus ASC/BSC.

Modelling and extrapolation of the clinical trial data were necessary to quantify the expected costs and benefits of the respective treatment groups. The economic analysis assessed the cost-effectiveness of cetuximab/irinotecan in-line with its licensed indication, for which there are no recognised comparator treatments established in clinical guidelines.

The primary health outcome for the economic evaluation is life-years gained (LYG); therefore, the primary health economic outcome is the incremental cost per life-year gained of cetuximab/irinotecan vs ASC/BSC. Secondary analyses were performed using quality adjusted life-years (QALYs) based on utility values directly observed from a separate clinical trial, the MABEL study, of cetuximab with irinotecan.

The economic evaluation estimated costs and consequences over the full lifetime of each patient. Modelling was required to extrapolate survival curves from the end of the follow-up period (Cunningham *et al*, 2004) until all patients were deceased.

Direct costs were estimated from the perspective a third-party payer (NHS); indirect and intangible costs were not included. Probabilistic and univariate sensitivity analyses were conducted for all model parameters in order to demonstrate the robustness of the results.

### Treatments compared

Published clinical evidence was evaluated and a survey of key medical oncologists in England and Wales was conducted in order to determine an appropriate comparator (i.e. the treatment displaced by irinotecan/cetuximab). This research investigation revealed that patients who fail second-line chemotherapy currently receive BSC; a minority will receive BSC with cytotoxic chemotherapy. We defined the treatment received by this late-stage CRC patient group as active/best supportive care (ASC/BSC). A key source of published evidence was identified in a trial of active chemotherapy (irinotecan) vs supportive care in the second-line setting (Cunningham *et al*, 1998). Clinical effectiveness and resource use data for the economic evaluation was taken from the clinical trials (Cunningham *et al*, 1998, 2004).

### Effectiveness estimates

Effectiveness estimates for the respective treatment groups were modelled from indirect clinical trial evidence (Cunningham *et al*, 1998, 2004). The modelling was undertaken, in the absence of head to head clinical trial data, in order to assess the incremental benefit of cetuximab/irinotecan compared to ASC/BSC.

### Overview of the estimation of survival benefit in the treatment and comparator groups

The best available evidence on the effectiveness of cetuximab/irinotecan in this setting is derived from Cunningham *et al* (2004). Calculation of the survival benefit of cetuximab/irinotecan therapy in the economic model requires modelling of Cunningham *et al* (2004) trial data for the cetuximab/irinotecan therapy arm. The economic evaluation compares cetuximab/irinotecan with the appropriate real-world comparator, ASC/BSC, rather than cetuximab monotherapy, which was applied in the study (Cunningham *et al*, 2004). Estimated overall survival benefit for patients receiving ASC/BSC had to be modelled using the effectiveness data from the comparator study (Cunningham *et al*, 1998) to account for the lower efficacy expected of ASC/BSC, where only a proportion of patients receive active treatment, compared with the cetuximab monotherapy patients, where all patients received an active treatment (Cunningham *et al*, 2004).

### Extrapolation of Cunningham *et al* (2004) censored survival data

Survival data for patients who participated in the pivotal cetuximab/irinotecan study (Cunningham *et al*, 2004) was imputed where necessary to account for censored values to allow calculation of overall life-expectancy in the two treatment arms, by estimating the missing tail-ends of the survival curves from the trial database (i.e. some patients were still alive at the end of the study, making it impossible to calculate average life-expectancy without some sort of imputation). Standard parametric methods and multiple imputations were used to formulate the estimates (Gelber *et al*, 1993; Parmar *et al*, 1998). In summary, survival estimates were separated into three stages:

#### First stage

The expected time for each censored observation was estimated by adding the predicted value from the parametric curve, conditioned on the individual's survival up to the censored time given that the individual has survived up until the censored time. The total estimated survival time was calculated as this quantity plus the censored time.

#### Second stage

Generation of random variables from the assumed tail distribution to impute the complete censored time. This was achieved by generating a survival probability from a uniform distribution of each censored observation; calculating where this probability would cut the time axis of the tail distribution conditional on the censored time; and adding this quantity to the censored time. This process is repeated 10 times, producing a ‘filled-in’ data set each time.

#### Third stage

Standard multiple imputation methods use each filled-in data set in a separate intended analysis and combine the required results based on the individual data sets.

Patients treated with cetuximab/irinotecan recorded an objective response rate (ORR) of 22.9%. (95% confidence interval (CI): 17.5–29.1) and a disease control rate (DCR) of 55.5% (95% CI: 48.6–62.2). The median overall survival of patients in the cetuximab/irinotecan group was 8.6 months in the trial (Cunningham *et al*, 2004).

### Estimation of survival benefit in the ASC/BSC comparator group

The current lack of direct comparative evidence for patients receiving ASC/BSC supports the extrapolation of treatment benefit based on the best available clinical evidence (Cunningham *et al*, 1998, 2004). Determination of an appropriate methodology to estimate survival benefit in the ASC/BSC treatment group was largely driven by the aim of maintaining the integrity of the clinical trial data from the pivotal study (Cunningham *et al*, 2004).

The methodological approach chosen to estimate the expected treatment benefit of cetuximab/irinotecan in the third-line setting used survival data for cetuximab monotherapy, together with the published and established relationship between active cytotoxic chemotherapy and ASC/BSC in the second-line setting. This trial (Cunningham *et al*, 1998) was appropriate for modelling because the comparator arm reflected ASC/BSC in current clinical practice (i.e. 31% of the patients in the supportive care arm received palliative chemotherapy).

The mean life-expectancy of patients receiving cetuximab monotherapy (Cunningham *et al*, 2004) was adjusted for censored patients and inclusion of LYG beyond the follow-up period.

This estimate of survival benefit was factored down to account for the expected treatment benefit conferred by active cytotoxic therapy. The factor applied was generated from clinical data (Cunningham *et al*, 1998). The relationship in the second-line setting between the survival benefit experienced by patients treated with active chemotherapy (irinotecan) and the survival benefit experienced by those treated with ASC/BSC was summarised in the form of a survival hazard ratio statistic as indicated by Table 1.

### Resource utilisation and cost data

Costs were calculated for the following resources:

- Study drugs received (cetuximab/irinotecan) and associated administration of drugs.
- Palliative chemotherapy for patients in the ASC/BSC arm.
- Nonchemotherapy resources while a patient is treated with cetuximab/irinotecan.
- Nonchemotherapy resources while a patient is treated with palliative chemotherapy in the ASC/BSC arm.
- Nonchemotherapy resources while a patient is not receiving any chemotherapy (in either arm).

Costs of study drugs and associated administration costs received by patients in the cetuximab/irinotecan arm were extracted from the pivotal trial (Cunningham *et al*, 2004). Owing to limitations in the literature detailing BSC costs in this patient population, resource utilisation estimates were extracted from 43 patients who were eligible for inclusion into the pivotal cetuximab study (Cunningham *et al*, 2004), but were not enrolled because recruitment had been completed (Data on File, UKECRC05019).

Patient resource utilisation data for chemotherapy, in-patient hospitalisations, outpatient consultations, laboratory tests (including EGFR testing) and imaging techniques were measured and costed for use in the model. Average weekly nonchemotherapy costs were calculated for patients treated with cetuximab/irinotecan, as well as for patients treated with other chemotherapies.

In summary, costs were accumulated in the economic model using the following methods:

- Cetuximab/irinotecan costs were calculated according to the amount of drug prescribed in the pivotal study (Cunningham *et al*, 2004). All patients had discontinued study drug by the end of the follow-up period.
- Patients treated with cetuximab/irinotecan accumulated administration costs of £255.54 per administration day, with the number of administration days determined from the pivotal cetuximab/irinotecan study (Cunningham *et al*, 2004).
- Patients treated with cetuximab/irinotecan accumulated the cost of a CT scan at 6 weeks (£49.01).
- Patients treated with cetuximab/irinotecan accumulated nonchemotherapy costs of £59.70 per week while they remained on treatment.
- Patients in the cetuximab/irinotecan treatment group accumulated nonchemotherapy costs of £50.00 per week after the disease progressed, and they were no longer on chemotherapy.
- A proportion of patients (31%) in the ASC/BSC group accumulated chemotherapy costs of £5327 per patient and associated administration costs of chemotherapy of £1482 per patient.
- Patients in the ASC/BSC group who received palliative chemotherapy accumulated nonchemotherapy costs of £50.00 per week until death.
- Patients in the ASC/BSC treatment group who did not receive palliative chemotherapy also accumulated nonchemotherapy costs of £50.00 per week until death.

## RESULTS

### Effectiveness estimates

**Figure 1:**
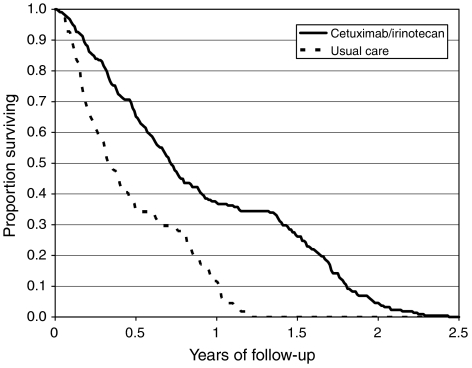
Modelled survival curves by treatment group.

The mean life-expectancy of patients receiving cetuximab/irinotecan, after adjustments for censored patients and inclusion of LYG beyond the follow-up period, was 11.01 months. Following adjustments for censored patients and inclusion of LYG beyond the follow-up period, the mean life-expectancy of patients receiving cetuximab monotherapy was 9.64 months (Cunningham *et al*, 2004). After factoring this value down to account for the added treatment effect of active cetuximab monotherapy, the mean estimated overall survival for patients receiving ASC/BSC was estimated to be 5.64 months (Table 2). Survival curves of the respective treatment groups estimated in the model are presented in Figure 1. The reduced life-expectancy of ASC/BSC patients is possibly due to the fact that only a minority of patients given ASC/BSC treatment in England and Wales received active cytotoxic therapy.

The discounted life-expectancy of patients treated with cetuximab/irinotecan was 0.91 life-years. This contrasted with 0.47 discounted life-years for patients receiving ASC/BSC. In summary, cetuximab/irinotecan was associated with an increase in survival of 0.44 discounted life-years per patient (Table 2).

A secondary analysis calculated the QALYs gained in each of the treatment groups. The separate cetuximab/irinotecan trial, the MABEL study, was used to estimate a utility value to apply in this analysis. The pivotal cetuximab/irinotecan study (Cunningham *et al*, 2004) did not collect indexed quality of life outcomes. The MABEL study collected utility values from 125 patients using the EQ-5D. The mean utility value of 0.746 estimated from MABEL was applied to all patients at all time points in the economic model. The estimated QALYs in the cetuximab/irinotecan and ASC/BSC treatment groups were 0.68 and 0.35, respectively.

### Cost estimates

The cost results for resource use in the two treatment groups are presented in Table 3. Over the duration of the model, patients treated with cetuximab/irinotecan accumulated mean additional costs of £18 901 per patient relative to ASC/BSC patients, of which £11 802 was attributable to cetuximab.

### Incremental cost-effectiveness

With additional costs of £18 901 per patient and LYG of 0.44 per patient, the incremental cost per life-year gained with cetuximab/irinotecan therapy compared with ASC/BSC was £42 975 (Table 4). The incremental cost per QALY gained was £57 608 (Table 4).

### Sensitivity analyses

Sensitivity analyses were undertaken to capture the statistical uncertainty of the costs and outcomes estimated in the model. An additional sensitivity analysis was conducted to determine which variables have the greatest influence on the results of the economic model.

**Figure 2:**
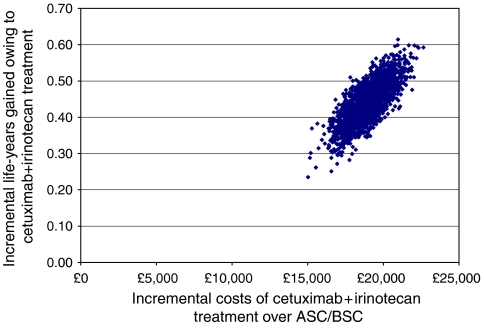
Scatter plot showing incremental costs of cetuximab/irinotecan over ASC/BSC.

A bootstrapping technique was applied to the individual patient-level data of the pivotal cetuximab/irinotecan study (Cunningham *et al*, 2004). The additional costs and LYG with cetuximab/irinotecan vs ASC/BSC for each of the 2000 bootstrap samples are plotted on the cost-effectiveness plane in Figure 2. This shows that cetuximab/irinotecan therapy was associated with additional life-years compared with ASC/BSC in all simulations of the model.

**Figure 3:**
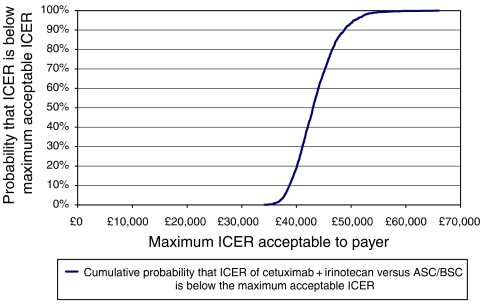
Cost-effectiveness acceptability curve based on 2000 bootstrap samples.

In order to derive a cost-effectiveness acceptability curve, the cumulative distribution of the incremental cost-effective ratios across the 2000 samples is presented in Figure 3. If, for example, £50 000 is accepted as a reasonable value for an additional life-year for an unmet clinical need, then it can be seen from Figure 3, that the likelihood of cetuximab/irinotecan being a cost-effective intervention is greater than 90%.

One-way sensitivity analyses were applied to the model in order to ascertain the cost-effectiveness of cetuximab/irinotecan in discrete scenarios. Variations in assumptions regarding costs and outcomes were examined in order to establish the strongest result drivers of the model and to verify the robustness of the primary results. Sensitivity analyses involving health outcome and cost variables are shown in Table 5. Of the health outcomes variables employed within the economic model, the cost-effectiveness of cetuximab/irinotecan is most sensitive to the survival adjustment factor. Of the various cost assumptions employed within the economic model, cost-effectiveness was most sensitive to the cost of cetuximab.

## DISCUSSION

Cetuximab in combination with irinotecan is licensed for the treatment of patients who have progressed after irinotecan therapy. In the UK, treatment with cetuximab/irinotecan is likely to be in the third-line setting, as irinotecan and oxaliplatin are currently recommended by NICE in the first- and second-line settings (NICE Technology Assessment no. 91, 2005). However, second-line usage may potentially be appropriate in a proportion of patients, for instance where oxaliplatin has been used adjuvantly and irinotecan used first-line. There are currently no standard therapeutic options for the third-line treatment of metastatic CRC, an area of unmet clinical need. Antibody therapy with cetuximab in combination with irinotecan offers a potentially effective therapeutic intervention for these patients. However, the routine introduction of such a treatment in this indication requires the demonstration of efficacy and cost-effectiveness compared with current treatment practice.

In this analysis, modelling was employed to extrapolate survival as a parameter to assess the cost-effectiveness of combination cetuximab/irinotecan therapy. Overall survival was not the primary end-point of the pivotal cetuximab/irinotecan study (Cunningham *et al*, 2004) and further definitive survival studies are underway. Different statistical approaches may be applied to survival modelling, and in the case of this analysis, involved the imputation of survival data for both treatment arms in the BOND study to account for censored values. Other statistical approaches, such as the Weibull technique, are feasible and all have inherent limitations. In the absence of survival data from a direct comparison of irinotecan in combination with cetuximab vs BSC/ASC, an indirect comparison and survival modelling was necessary. Although this poses some drawbacks, the use of a single randomised controlled trial as a vehicle for economic analysis also has limitations and may lead to a partial and limited analysis to inform decision making. Furthermore, the health-economic parameters required for cost-effectiveness analyses, such as health-related utilities, treatment effects, resource use and costs, are often absent or highly uncertain from early clinical trials of new chemical entities. The most appropriate framework for economic analysis is evidence synthesis and decision modelling, where all available data impacts upon the specific decision problem and may sometimes involve indirect comparisons (Sculpher and Claxton, 2005). In addition, new treatments in oncology offer particular methodological problems for cost-effectiveness analyses as (i) new agents often add a new element in a sequential treatment pathway and (ii) the standard comparator is either absent or highly variable across treatment centres and national healthcare systems.

The choice of the comparator for the indirect comparison was drawn from a trial of irinotecan vs supportive care in patients failing first-line 5-FU-based chemotherapy (Cunningham *et al*, 1998). This was selected principally, as the supportive care arm represented the appropriate group for the comparator of ASC/BSC where 31% of the patients had received ASC. Two other trials of chemotherapy in the second/subsequent-line treatment vs supportive care for of metastatic CRC (Barni *et al*, 1995; Rao *et al*, 2004) were considered inappropriate proxies in this analysis as the supportive care arms comprised BSC alone and no ASC. Of note, survival figures differed between these studies such that use of alternative studies may have led to different cost-effectiveness outcomes such as cost per QALY. However, caution should be exercised in such cross-study comparisons and implications for cost-effectiveness owing to trial heterogeneity and differences in baseline patient characteristics. A full discussion of the potential pitfalls of the various methodologies that can be employed in this context is beyond the scope of this article, but is well documented elsewhere (http://nice.org.uk/page.aspx?o=325816).

The incremental cost per life-year gain of £42 975 for cetuximab/irinotecan is relatively high compared with other health-care interventions. However, this result should be considered in the context of a number of factors specific to this therapy and patient population, with limited therapeutic options and a generally low life-expectancy. The economic model showed that cetuximab/irinotecan nearly doubles patient life-expectancy (0.91 life-years vs 0.47 life-years). Camidge *et al* (2005) have recently proposed that the proportion of life-saved should be a consideration in decision making – over and above the absolute level of life-saved. Many disease types shorten life to a greater or lesser extent; if an expensive new treatment allows a terminal cancer patient to live 3 months longer, then it seems inappropriate that this should be ascribed the same low value-for-money rating (i.e. cost per QALY threshold) as a treatment that provides the same incremental survival benefit in the context of a chronic condition [Camidge *et al*, 2005]. Similar arguments have previously been proposed by others (Brouwer and van Hout, 1998; Waugh and Scott, 1998). For patients with a poor prognosis, the absolute level of life-saved will likely be relatively low. The concept of ascribing higher cost-effectiveness thresholds to patients with lower life-expectancy is consistent with the ‘rule of rescue’, which applies greater value to therapies for patients with poor prognosis and few available alternatives and which are life-prolonging.

The incremental cost per QALY gained estimated in this analysis (£57 608) is also relatively high. Again, this result should be interpreted in the context of the patient population as described in the previous paragraph, but also in terms of the applicability of utility values to this patient population. It is not clear that the EQ-5D instrument does not captures the value that patients apply to the final stages of their life. In a utility study using the time-trade-off technique, Petrou *et al*. estimated a utility value of 0.95 in metastatic CRC patients (Petrou and Campbell, 1997). Although this value does not necessarily reflect quality of life *per se*, it does capture the value patients place on their remaining life-expectancy. This is an important consideration for decision makers which cannot be included in the economic analysis.

With the preceding discussion in mind, and the fact that in the UK cancer survival is an established national health priority (NHS Cancer Plan), it is reasonable to consider accepting higher levels of cost-effectiveness for cetuximab/irinotecan in this patient group. Traditionally, an incremental cost per life-year gained of £25 000–£30 000 has been deemed an acceptable threshold (Rawlins and Culyer, 2004). If this threshold was increased to £50 000 for specific diseases, or even specific stages of disease, then our analysis shows that cetuximab/irinotecan would represent value for money. A recent publicly funded research venture, which sought to estimate the monetary value of a QALY based upon values applied in other areas of UK government policy, noted that QALY gains for life saving/extending health interventions should use a value of between £45 000 and £60 000 per QALY (Mason *et al*, 2006).

In the case of cetuximab, strategies aimed at lowering the incremental cost per life year gained or QALY have previously been considered with the specific aim of targeting patients more likely to benefit from therapy. An association between the presence and intensity of skin rash and longer survival with cetuximab-based therapy has been documented (Cunningham *et al*, 2004; Perez Soler and Saltz, 2005). In one model, the use of an early CT scan at 6 weeks could be used to stratify patients for further treatment; responders would continue therapy, those with progressive disease would cease therapy and those with stable disease would continue therapy only if they had developed skin rash, the so-called continuation rule. Such a strategy would inevitably reduce the economic impact of treatment. However, disease stabilisation with cetuximab/irintotecan even in the absence of skin rash is also an important outcome, particularly in the face of irinotecan-refractory disease. For the purpose of this analysis, a continuation rule was not applied. It should also be noted that currently there are no other validated clinical or biological markers by which to select patients most likely to respond to cetuximab, although several studies are underway to explore this area further. No correlation has been demonstrated between the presence or intensity of EGFR expression and response to therapy (Cunningham *et al*, 2004). There have also been reports of response to cetuximab/irinotecan in patients whose tumours do not express EGFR (Chung *et al*, 2005). The identification and validation of an appropriate predictive marker would help to enrich the responding population and potentially increase cost-effectiveness, which applies equally to other emerging ‘targeted’ drugs.

## CONCLUSION

The therapeutic landscape of metastatic CRC has significantly changed over the last decade with the advent of new cytotoxic drugs. Their integration with new biologic agents represents an important scientific and clinical advance and heralds a new treatment era for the disease. The optimal means by which to integrate these drugs will continue to evolve, as data emerges from a multitude of studies that are currently underway. In order that patients may benefit from current advances in a timely manner, physicians, patient groups and health commissioners need to coordinate their assessments and decision-making criteria to determine the optimal usage for NHS patients of new drugs such as cetuximab.

## Figures and Tables

**Table 1:** Estimating survival in the ASC/BSC treatment group

**Row**	**Parameter**	**Value**	**Reference**
A	Mean survival in patients treated with cetuximab monotherapy in the BOND study	9.64 months	Analysed using individual patient data, including adjustments for censored patients
			
B	Survival hazard ratio for irinotecan-treated patients relative to best supportive care patients in a second-line setting	1.71	Cunningham *et al* (1998)
			
C	Survival hazard ratio for cetuximab monotherapy-treated patients relative to best supportive care patients	1.71	Assumption that effect of cetuximab monotherapy in the third-line setting will be the same as irinotecan treatment effect in the second-line setting (as observed, row B)
			
D	Mean survival in patients in ‘usual care’ for the patients treated with cetuximab monotherapy in the BOND study	5.64 months	A/C. Netting out the cetuximab monotherapy effect in row C from the monotherapy cetuximab survival observed in BOND (row A)

**Table 2:** Life-expectancy of patients in the economic evaluation, by treatment group

**Treatment group**	**Life-expectancy (undiscounted)**	**Reference**	**Life years gained (discounted^a^)**	**Incremental life years gained of cetuximab/irinotecan**
Cetuximab/irinotecan	11.01 months	Cunningham *et al* (2004) with after adjustments for censored patients	0.9120	
				
Cetuximab monotherapy	9.64 months	Cunningham *et al* (2004) with after adjustments for censored patients	0.8003	0.1116
				
ASC/BSC	5.64 months	Cetuximab monotherapy with adjustment for treatment effect based on Cunningham *et al* (1998) (5.64=9.64/1.71)	0.4722	0.4398

a Life years are discounted at 3.5% per annum consistent with NICE recommendations.

**Table 3:** Total costs by treatment group estimated in the economic model

**Resource item**	**Cetuximab/ irinotecan**	**ASC/BSC**	**Incremental**
Cetuximab costs	£11 802	£0	£11 802
Irinotecan costs	£3593	£0	£3593
Other chemotherapy costs	£0	£1680	−£1680
Administration costs	£4300	£467	£3832
Other resource costs	£2574	£1221	£1353
			
Total costs	£22 270	£3368	£18 901

**Table 4:** Incremental cost-effectiveness ratios estimated in the model

**Treatment group**	**Total costs**	**Life-years gained**	**Incremental cost per life-year gained**	**QALYs gained**	**Incremental cost per QALY gained**
Cetuximab/irinotecan	£22 270	0.9120		0.6803	
ASC/BSC	£3368	0.4722		0.3522	
Incremental	£18 901	0.4398	£42 975	0.3281	£57 608

**Table 5:** Incremental cost-effectiveness of cetuximab/irinotecan over ASC/BSC resulting from one-way sensitivity analyses involving health outcomes variables

**Description of sensitivity analysis**	**Value of variable/assumption in base case**	**Value of variable/assumption in sensitivity analysis**	**Incremental costs**	**Incremental life-years gained**	**Incremental cost per life-year gained**
Base case	—	—	£18 901	0.4398	£42 975
Approximate proportion of ASC/BSC patients who receive chemotherapy	31%	20%	£19 638	0.4398	£44 649
Approximate proportion of ASC/BSC patients who receive chemotherapy	31%	40%	£18 288	0.4398	£41 581
Survival adjustment factor to BOND monotherapy data in estimation of ASC/BSC survival	1.71	1.50	£18 732	0.3747	£49 999
Survival adjustment factor to BOND monotherapy data in estimation of ASC/BSC survival	1.71	2.00	£19 078	0.5076	£37 587
Cost of cetuximab vial	£136.50	£90.00	£14 881	0.4398	£33 834
Cost of chemotherapy administration	£255.54	£500	£22 568	0.4398	£51 311
Cost of chemotherapy administration	£255.54	£50	£15 819	0.4398	£35 967
Cost of BSC while receiving cetuximab/irinotecan (weekly)	£59.70	£100	£19 602	0.4398	£44 568
Cost of BSC while receiving cetuximab/irinotecan (weekly)	£59.70	£1	£17 881	0.4398	£40 656
Cost of BSC while receiving other chemotherapy (weekly)	£50.00	£100	£19 174	0.4398	£43 596
Cost of BSC while receiving other chemotherapy (weekly)	£50.00	£1	£18 634	0.4398	£42 367
